# Pterostilbene Acts through Metastasis-Associated Protein 1 to Inhibit Tumor Growth, Progression and Metastasis in Prostate Cancer

**DOI:** 10.1371/journal.pone.0057542

**Published:** 2013-03-01

**Authors:** Kun Li, Steven J. Dias, Agnes M. Rimando, Swati Dhar, Cassia S. Mizuno, Alan D. Penman, Jack R. Lewin, Anait S. Levenson

**Affiliations:** 1 Cancer Institute, University of Mississippi Medical Center, Jackson, Mississippi, United States of America; 2 United States Department of Agriculture, Agricultural Research Service, Natural Products Utilization Research Unit, University, Mississippi, United States of America; 3 Center of Biostatistics, University of Mississippi Medical Center, Jackson, Mississippi, United States of America; 4 Department of Pathology, University of Mississippi Medical Center, Jackson, Mississippi, United States of America; University of Kentucky College of Medicine, United States of America

## Abstract

The development of natural product agents with targeted strategies holds promise for enhanced anticancer therapy with reduced drug-associated side effects. Resveratrol found in red wine, has anticancer activity in various tumor types. We reported earlier on a new molecular target of resveratrol, the metastasis-associated protein 1 (MTA1), which is a part of nucleosome remodeling and deacetylation (NuRD) co-repressor complex that mediates gene silencing. We identified resveratrol as a regulator of MTA1/NuRD complex and re-activator of p53 acetylation in prostate cancer (PCa). In the current study, we addressed whether resveratrol analogues also possess the ability to inhibit MTA1 and to reverse p53 deacetylation. We demonstrated that pterostilbene (PTER), found in blueberries, had greater increase in MTA1-mediated p53 acetylation, confirming superior potency over resveratrol as dietary epigenetic agent. In orthotopic PCa xenografts, resveratrol and PTER significantly inhibited tumor growth, progression, local invasion and spontaneous metastasis. Furthermore, MTA1-knockdown sensitized cells to these agents resulting in additional reduction of tumor progression and metastasis. The reduction was dependent on MTA1 signaling showing increased p53 acetylation, higher apoptotic index and less angiogenesis *in vivo* in all xenografts treated with the compounds, and particularly with PTER. Altogether, our results indicate MTA1 as a major contributor in prostate tumor malignant progression, and support the use of strategies targeting MTA1. Our strong pre-clinical data indicate PTER as a potent, selective and pharmacologically safe natural product that may be tested in advanced PCa.

## Introduction

Prostate cancer (PCa) treatment still represents an unmet medical need. Diet-derived polyphenols, including stilbenes, are attractive clinical candidates for primary and secondary cancer chemoprevention due to their ability to not only block or inhibit initiation of carcinogenesis but also to reverse the promotional stages. The latter characteristic makes these compounds promising therapeutic agents. Resveratrol (Res) and other natural stilbenes are phytoalexins that are produced by plants in response to environmental stress [1]. Resveratrol has cardioprotective, anti-inflammatory and anticancer activities [2], [3]. The anticancer actions of resveratrol involve regulation of multiple and diverse molecular targets and are mediated by induction of cell cycle arrest and apoptosis [4]–[8] and inhibition of angiogenesis [9]–[13].

We recently discovered that resveratrol downregulates metastasis-associated protein 1 (MTA1) in PCa [13]. MTA1 overexpression is correlated with clinicopathological parameters that characterize tumor aggressiveness: lymph node metastasis, high tumor grades, and angiogenesis in various cancers [14]–[19]. In human prostate tissues, a high MTA1 level was associated with hormone-refractory PCa [15]. We initially identified MTA1 as a novel participant in the “vicious cycle” of PCa bone metastasis, and further demonstrated that intensity of staining and nuclear localization of MTA1 in human tissues were correlated with the aggressiveness of PCa and bone metastatic lesions [19]. We also established that MTA1 had pro-survival, anti-apoptotic, invasive and pro-angiogenic properties in PCa *in vitro* and *in vivo* [19].

MTA1 is a part of the nucleosome remodeling and deacetylation (NuRD) co-repressor complex involved in histone and non-histone protein deacetylation and gene-specific transcriptional regulation [20], [21]. The complex also contains histone deacetylases 1 and 2 (HDAC1 and 2). We previously showed that resveratrol-induced MTA1 degradation destabilizes the MTA1/HDAC/NuRD deacetylation complex leading to increased acetylation/activation of tumor suppressor p53 in PCa cells [13]. MTA1 silencing through RNAi significantly sensitized the PCa cells to resveratrol-dependent p53 acetylation and apoptosis. This HDAC inhibitor-like activity of resveratrol was further supported by combination experiments with clinically approved HDAC inhibitor suberoylanilide hydroxamic acid (SAHA), in which resveratrol synergistically enhanced p53-acetylation and apoptosis [13]. We have also shown that MTA1 knockdown cells had suppressed invasion and angiogenic activity *in vitro*, and delayed tumor formation and development in subcutaneous xenografts [19]. Taken together, we defined a novel epigenetic mechanism of anticancer activity of resveratrol mediated through the negative epigenetic regulator, MTA1.

Despite its promising properties, resveratrol’s rapid metabolism and low bioavailability have precluded its advancement to clinical use [2], [22]–[24]. The limitations of resveratrol have prompted our interest in natural and synthetic analogues with improved pharmacokinetics and superior pharmacological potency that hold greater potential as anticancer natural product drugs [25]. However, how resveratrol analogues compare with each other in terms of potency and mechanisms of action is largely unknown. The *in vivo* effects are mainly unexplored for resveratrol analogues, which makes it difficult to select which analogue(s) is the best for clinical development.

In the current study, we carried out a comparative analysis of resveratrol and six analogues in their ability to inhibit MTA1 expression and signaling, and focused on MTA1-mediated anticancer and antimetastatic effects of the most potent analogue, PTER, using orthotopic PCa xenografts. Our results indicate MTA1 as a powerful target for intervention by dietary PTER as therapeutic natural product drug for anticancer therapy in primary and metastatic PCa.

## Materials and Methods

### Ethics Statement

All animal work was conducted according to approved animal protocol #1272 by the Institutional Animal Care and Use Committee of University of the Mississippi Medical Center and accredited by the Association for Assessment and Accreditation of Lab Animal Care International in compliance with the US Public Health Service policy as assured by the Office of laboratory Animal Welfare.

### Materials

Resveratrol and piceatannol (PIC) were obtained from commercial sources (Sigma-Aldrich, St. Louis, MO and Calbiochem-Novabiochem Corp., San Diego, CA, respectively). Pterostilbene was synthesized following published procedures [26] Trimethoxy-resveratrol (3M-Res) and (*E*)-4-(3,5-dimethoxystyryl)aniline (DMSA) were synthesized according to previously described methods [27]. Triacetyl-resveratrol (3Ac-Res) and 3,5-diacetyl resveratrol (2Ac-Res) were synthesized as follows: to 18 mg of resveratrol dissolved in 500 µL of MeOH, 10 drops of pyridine and 500 µL of acetic anhydride were added. The mixture was stirred overnight at room temperature. Ethyl acetate (EtOAc) was added to the reaction mixture, and then partitioned with water to remove pyridine. The EtOAc layer was concentrated under a stream on nitrogen, and spotted on a thin-layer chromatography plate. The plate was developed using solvent system 30% EtOAc:70% hexane. The bands of 3Ac-Res (Rf 0.8) and 2Ac-Res (Rf 0.6) were scraped from the plate and extracted with EtOAc. The structures of 3Ac-Res and 2Ac-Res were determined by nuclear magnetic resonance and mass spectrometry. The structures of all other stilbenes have also been confirmed by spectroscopy. The purity of the compounds was determined to be >99%. The compounds were dissolved in high purity ethanol or DMSO and stored in the dark at −20°C prior to use in assays.

### Cell Culture

LNCaP and Du145 PCa cells were purchased from American Type Culture Collection (ATCC). PC3M cells were a gift from Dr. R. Bergan (Northwestern University, Chicago, IL) [28], [29], and RWPE-1, “normal” immortalized prostate epithelial cells, were a gift from Dr. C. Lee (Northwestern University, Chicago, IL) [30]. PCa cells were grown in RPMI-1640 containing 10% FBS, 5% penicillin/streptomycin as previously described [13], [19]. RWPE-1 cells were grown in ATCC complete growth medium, which contain base keratinocyte serum free medium supplemented with bovine pituitary extract and human recombinant epidermal growth factor. Cells were maintained in an incubator at 37°C with 5% CO_2_. Media was replaced with phenol red-free RPMI-1640 with 5% charcoal-stripped serum 16–18 hr prior to resveratrol/analogues treatments to provide steroid-free background.

### Western Blot Analysis

Western blot analysis was done as described previously [13], [19]. Briefly, cells were treated with various concentrations of resveratrol or analogues for 24 hr. The cells were washed with cold PBS, and lysed by RIPA lysis and extraction buffer containing Halt Phosphatase and Protease Inhibitor Cocktail (Thermo Scientific). The protein concentration was measured using the Bio-Rad protein assay reagent (Bio-Rad Laboratories, Hercules, CA). Equal amounts of protein (40–70 µg) were resolved in 7–10% Tris-TGX Ready gels and transferred to a PVDF membrane by Mini Trans-Blot Electrophoresis Transfer System (Bio-Rad Laboratories, Hercules, CA). The membranes were then blocked with TBS-Tween and 5% dry milk for 2 hours at room temperature and probed with MTA1, AR, p53 (Santa Cruz Biotechnology, Santa Cruz, CA) or Ac-p53(K381) (Abcam) antibodies. The blots were then probed with β-actin antibodies (Sigma-Aldrich, St. Louis, MO) as a loading control. Signals were visualized using enhanced chemiluminescence. Densitometry was done using Image J. Effective dose (ED_50_) values were calculated by the Chou-Martin method using CompuSyn for Drug Combinations and for General Dose-Effect Analysis software (ComboSyn, Inc., Paramus, NJ).

### Generation of Stable Cells Tagged with Luciferase

MTA1-knockdown Du145 cells were previously established using the Expression Arrest GIPZ lentiviral shRNAmir vectors expressing MTA1shRNA or non-silencing empty vector (EV, Open Biosystems) and were characterized *in vitro* and *in vivo* [13], [19]. Vectors contained green fluorescent protein (GFP) and a puromycin resistance gene. Although cells demonstrated high GFP signals *in vitro*, the *in vivo* experiments were unsuccessful due to high background signals from the animal tissues. As bioluminescence has high signal-to-noise compared to fluorescence, we transduced cells with the lentiviral luciferase (Luc) construct (a gift from Dr. R. Graeser, Department of Medical Oncology, Freiburg, Germany) and selected stable clones using puromycin. Clonal populations of cells were expanded *in vitro* and tested for their luciferase activities (see below). Several bright clones were repeatedly selected again and pooled together for cells with high luciferase expression to be used *in vivo.*

### *In vitro* and *in vivo* Bioluminescent Imaging

*In vitro* and *in vivo* bioluminescence (BL) was performed using an IVIS Spectrum (Caliper Life Sciences, Hopkinton, MA). Images and detection of BL signals were acquired and analyzed using Living Image Software V. 4.1. For *in vitro* imaging, cells tagged with luciferase were serially diluted in a black, clear bottom 96-well plate (Costar, Corning, NY). D-luciferin (150 µg/ml) was added to the wells, the plate was incubated at 37°C, 5% CO_2_ for 10 min after which images were taken. For *in vivo* BL imaging, anesthetized mice were injected intraperitoneally (i.p.) with 150 mg/kg D-luciferin (Caliper) and placed inside the camera box for 10 min. Continuous exposure to 2% isoflurane sustained sedation during imaging. Acquisition time was usually maximum 3 min with auto-exposure depending on the BL of tumors. Measurements of emitted light were performed for regions of interest (ROI) and quantified as photon flux (photons/sec/cm2/sr). Normalization was done for all images at the end of the experiment. Gray scale images of mice were also collected at each session. Detection of metastasis during the experiment was contaminated by strong primary signal resulting in false-positive signals. Therefore, at the end of experiment, we secluded the primary signal by excising the prostate and surrounding muscle tissue to expose organs in abdominal cavity. Kidneys, livers and lung/heart from each mouse were isolated and *ex vivo* imaged by increasing binning and F/stop.

### Orthotopic PCa Xenografts

Seven-week-old male nude mice (Fox n1nu, Harlan) were fed phytoestrogen-free AIN-76A diet from the day received. Animals were randomly assigned to two major groups prior to surgery and subsequently injected with either Du145-EV-Luc (EV) or Du145-MTA1shRNA-Luc (MTA1shRNA) cells. During the surgery mice were anesthetized with 2% isoflurane, a 7–9 mm abdominal midline incision was made, and the prostate was exposed. 2.5×10^6^ cells of each cell line, in 20 µl PBS and Matrigel, were injected into anterior prostate. The wound was sutured, and the skin was closed with autoclips. Animals were closely monitored after surgery and clips were removed at day 10. Tumors colonized and grew for 14 days. Mice that developed tumors in each group were randomized into three subgroups (n = 6) with daily i.p. administration of 10% DMSO sham control (Ctrl); 50 mg/kg resveratrol or PTER. Both compounds were soluble in 10% DMSO when warmed-up using water bath. Each week fresh solutions were prepared for injections. Body weights and BL signals were monitored weekly. The mice were sacrificed at week 8 after cell inoculation. At necropsy, prostates and other organs were excised, *ex vivo* imaged and fixed with 10% neutral-buffered formalin (Richard Allan Scientific, Kalamazoo, MI). Serum was also collected and stored at −80°C.

### Histopathology and Immunohistochemistry

Four µM thick sections were prepared from formalin-fixed paraffin embedded tumors and stained with hematoxylin and eosin (H&E) using standard protocol. Immunohistochemistry (IHC) was applied to evaluate Ki-67, p53, Ac-p53 (K381), M30 and CD31 as previously described [19]. Briefly, sections were deparaffinized, rehydrated with xylene and descending grades of alcohol and water. Antigen retrieval was performed by boiling the slides in Antigen Unmasking Solution (Vector Labs, CA) for 30 min in a steamer. Slides were cooled and endogenous peroxidase activity was quenched in 3% H_2_O_2_ in ethanol for 5 min. Blocking was performed in accordance with the appropriate antibodies employed for staining using the Vectastain ABC Elite Kit (Vector labs, CA). Sections were incubated overnight at 4°C with the following antibodies: anti-Ki67 (1∶100) and anti-p53 (1∶100, Santa Cruz Biotechnologies, CA), anti-CD31 (1∶100) and anti-Ac-p53 (1∶100, Abcam, MA) and anti-M30 (1∶100, Roche Diagnostics, GmBH, Germany). Sections were washed and incubated with appropriate secondary antibodies from the ABC kit and staining was revealed using the ImmPACT DAB kit (Vector Labs, CA). Slides were counterstained in hematoxylin, dehydrated and mounted. Images were viewed and recorded on Nikon Eclipse E400 microscope. The ImageTool software was used to count positively-stained cells in five randomly selected fields.

### Analysis of Resveratrol and Pterostilbene Content in Murine Serum by Gas Chromatography-mass Spectrometry (GC-MS)

Serum, stored in −80°C freezer prior to use, was centrifuged at 4°C for 15 min and treated with 80 µL of β-glucuronidase (1250 U/mL potassium phosphate buffer 75 mM, pH 6.8 at 37°C). The mixture was incubated at 37°C with shaking at 750 rpm, for 17.5 hrs, cooled to room temperature then partitioned with 75 µL EtOAc. The EtOAc layer was dried under a stream of nitrogen, derivatized with 30 µL of 1∶1 mixture of N,O-bis[trimethylsilyl]trifluoroacetamide and dimethylformamide (Pierce Biotechnology, Inc., Rockford, IL) and used for the analysis. GC-MS analysis was performed using a JEOL GCMate II Instrument (JEOL, USA Inc., Peabody, MA) with a J&W DB-5 capillary column (0.25 mm internal diameter, 0.25 µm film thickness, 30 mm length; Agilent Technologies, Foster City, CA). The GC temperature program was: initial 190°C held for 1 min, increased to 244°C at the rate of 30°C/min and held at this temperature for 0.5 min, increased to 246°C at the rate of 0.5°C/min and held for 0.5 min, increased to 280°C at the rate of 20°C/min and held for 2 min, then finally increased to 300°C at the rate of 30°C/min and held for 0.8 min. The carrier gas was ultrahigh purity helium, at 1 mL/min flow rate. The injection port was kept at 250°C, the GC-MS interface at 230°C, and the ionization chamber at 230°C. The volume of injection was 2 µL (splitless injection). The mass spectrum was acquired in selected ion-monitoring mode, electron impact 70 eV. Pterostilbene was monitored with m/z 328, 313 and 297 (retention time 11.6 min). Resveratrol was monitored with m/z 444, 428 and 414 (retention time 13.7 min). Quantitation was done using external standards of a commercial sample of resveratrol (Sigma-Aldrich, St. Louis, MO) and a synthetic sample of PTER [31].

### Statistical Analyses

Values are expressed as the mean±SE or box plots of measurements. Student’s one-tailed paired t-test was used to analyze *in vitro* data. Differences in tumor BL intensity *in vivo* were analyzed by either rank-based or two-way ANOVA followed by Bonferroni post hoc analysis or by Kruskal-Wallis test. For some analysis data values were log transformed. The differences were considered significant at p<0.05.

## Results

### Pterostilbene is a Potent Inhibitor of MTA1 Expression in PCa Cells

**Figure 1:**
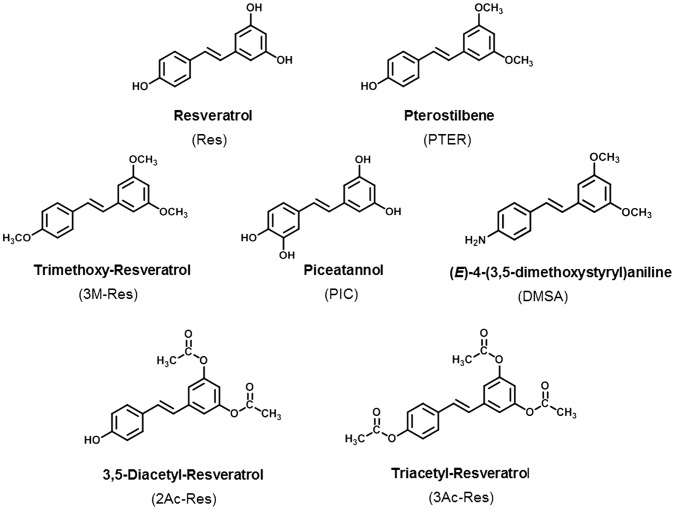
Chemical structures of stilbenes. Resveratrol (Res), *trans*-3,5,4′-trihydroxystilbene; Pterostilbene (PTER), *trans*-3,5-dimethoxystilbene; Trimethoxy-Resveratrol (3M-Res), *trans*-3,5,4′-trimethoxystilbene; Piceatannol (PIC), *trans*-3,5,3′4′-tetrahydroxystilbene; Dimethoxystyrylaniline (DMSA), *trans*-4-(3,5-dimethoxystyryl)aniline; Diacetyl-Resveratrol (2Ac-Res), *trans*-3,5-diacetylstilbene; Triacetyl-Resveratrol (3Ac-Res), *trans*-3,5,4′-triacetylstilbene.

**Figure 2:**
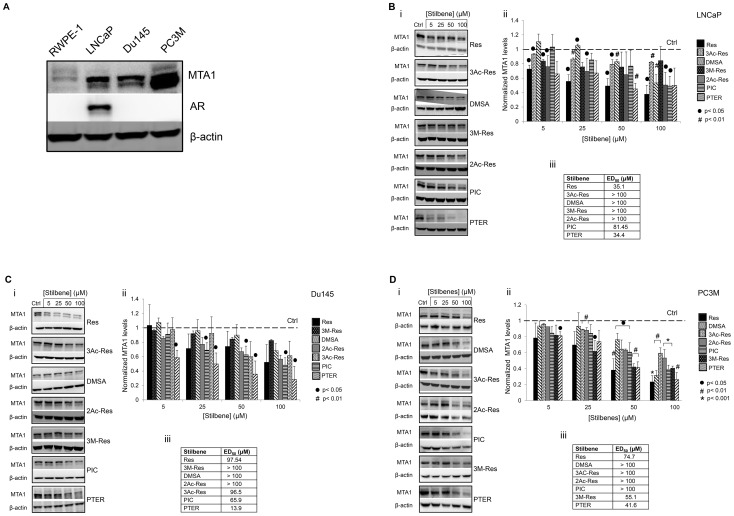
Differential expression of MTA1 in PCa cells analyzed by Western blot. A. Cell lines represent different stages of PCa progression: RWPE-1, “normal” immortalized prostate epithelial cells; LNCaP, androgen-responsive cells; Du145, androgen-resistant cells; PC3M, aggressive metastatic cells. Cells were grown in RPMI-1640 media containing 10% FBS and antibiotics. 75 µg of protein was separated on 10% gel, transferred to membrane and probed with MTA1 antibodies. β-actin was a loading control. **Pterostilbene has the highest potency in inhibiting MTA1.** B. Dose-dependent effects of Res/analogues on MTA1 protein levels in LNCaP; in Du145 (C ) and in PC3M (D) cells. (i) cells were treated with 5–100 µM of Res/analogues for 24 hr and analyzed by immunoblotting. (ii) graphical representation of results. The Ctrl is set to 1 and MTA1 level changes are expressed as a percentage of Ctrl. The means±SE of four independent experiments are shown. •p<0.05, #p<0.01, *p<0.001. (iii) effective doses (ED_50_) were calculated by Chou-Martin method.

**Figure 3:**
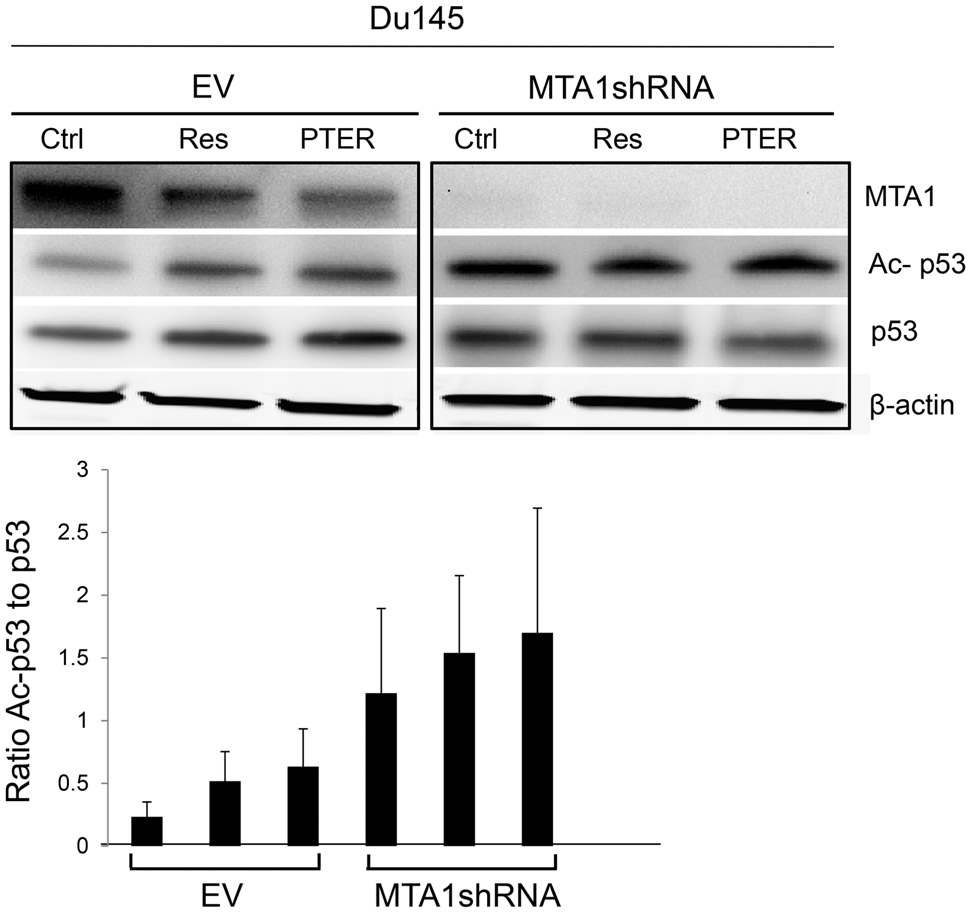
Pterostilbene increases MTA1-mediated p53 acetylation in Du145 cells. Du145-EV and Du145-MTA1shRNA cells (Fig. S1) were treated with 50 µM of Res or PTER for 24 hr and analyzed for MTA1, p53, Ac-p53 by Western blot as described in “Materials and methods”. A representative blot is shown. Quantitation of Ac-p53/p53 ratio was conducted by Image J software and data shown as mean±SEM from three independent experiments.

We recently discovered that resveratrol inhibits the epigenetic modifier MTA1, which ultimately leads to increased p53 acetylation and apoptosis of PCa cells [13]. Here, we tested six resveratrol analogues (Fig. 1) to determine whether they would have better anticancer activity through inhibition of this specific molecular target. Of the analogues, PTER, piceatannol (PIC) and trimethoxy-resveratrol (3M-Res) are naturally-occurring while dimethoxystyrylaniline (DMSA), diacetylstilbene (2Ac-Res) and triacetylstilbene (3Ac-Res) are synthetic derivatives. We examined the effects of these analogues on MTA1 expression in three PCa cell lines, representing different stages of PCa progression. The cells express MTA1 at different levels, from moderately high in androgen responsive LNCaP and androgen-resistant Du145 to very high levels in metastatic PC3M cells (Fig. 2A). We treated the cells with different doses (5–100 µM) of resveratrol/analogues for 24 hr and isolated protein for Western blots. There was down-regulation of MTA1 expression in compound-treated compared to vehicle-treated cells (Fig. 2). Resveratrol and analogues inhibited MTA1 protein levels in a dose-dependent manner but with different potencies. MTA1-inhibition by analogues was cell-specific: while the inhibitory effect of PTER was comparable with that of resveratrol in LNCaP cells (Fig. 2B), in Du145 cells, 3Ac-Res, PIC and PTER were more potent than resveratrol, but only PTER had an ED_50_ value in the low micromolar range (13.9 µM) (Fig. 2C). In PC3M cells, 3M-Res and PTER demonstrated higher potency than resveratrol, and once again, PTER had the lowest ED_50_ value (41.6 µM; Fig. 2D). Therefore, among the six analogues tested, PTER demonstrated the most potent MTA1-inhibition across all three cell lines. Pterostilbene was initially isolated from sandalwood and later was found in grapes and blueberries [31], [32]. Like resveratrol, PTER is a potent antioxidant and anti-inflammatory agent with chemopreventive and anticancer activity [33]–[42]. In Du145 cells, PTER exhibited the highest MTA1-inhibitory potency (more than seven times higher than resveratrol). Importantly, PTER demonstrated greater increase in MTA1-mediated p53 acetylation, especially in MTA1-knockdown sensitized cells (Fig. 3), implying superior potency over resveratrol as dietary epigenetic agent that controls posttranslational modifications of proteins.

### Effects of Resveratrol and Pterostilbene on Orthotopic Tumor Growth, Progression and Metastasis: Involvement of MTA1

We have previously shown that endogenous levels of MTA1 promote tumor development in a subcutaneous model of PCa [19]. However, the role of MTA1 in PCa growth, progression and metastasis remains unknown and the effects of resveratrol and PTER have not been evaluated in orthotopic PCa models. To compare the *in vivo* efficacy of resveratrol and PTER, and to study the role of MTA1 in prostate tumor growth and progression, we utilized clinically relevant orthotopic mouse model, in which human PCa cells expressing or silenced for MTA1 can grow in an environment related to their tissue origin. The goal of this experiment was three-fold: 1) to evaluate efficacy of resveratrol and PTER in inhibiting orthotopic PCa growth and progression; 2) to assess the role of MTA1 in prostate tumor development, progression and metastasis, and 3) to determine whether MTA1 affects the susceptibility of primary prostate tumor and metastasis to resveratrol and PTER.

**Figure 4:**
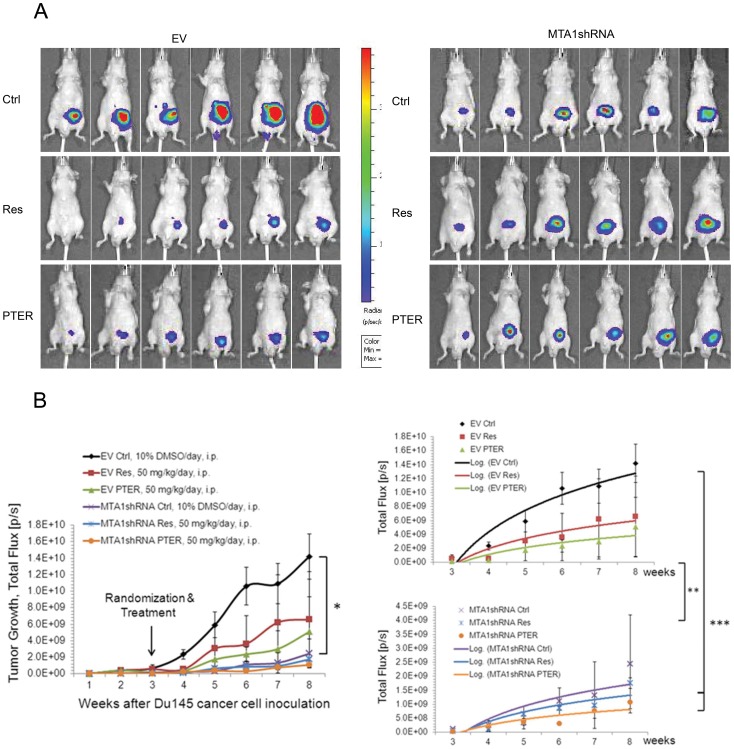
MTA1-mediated therapeutic activity of resveratrol and pterostilbene in orthotopic PCa xenografts. Male nude mice were injected orthotopically with Du145-EV-Luc (EV) or Du145-MTA1shRNA-Luc (MTA1shRNA) cells and treated with vehicle (Ctrl), Res or PTER, 50 mg/kg/day, every day, i.p. A. Normalized representative BL images of mice from each group are shown. B, *left*, Quantitative analysis of tumor light emission in Total Flux (photons/sec/cm2/sr) is plotted against time. The means ± SE are shown (n = 6 at the start), *p = 0.05. *Right*, log trends for each group are shown. Significant growth inhibition was detected in EV- vs. MTA1shRNA-tumors as groups, **p<0.01 and between EV-Ctrl vs. MTA1shRNA-Res and MTA1shRNA-PTER, ***p<0.001.

**Figure 5:**
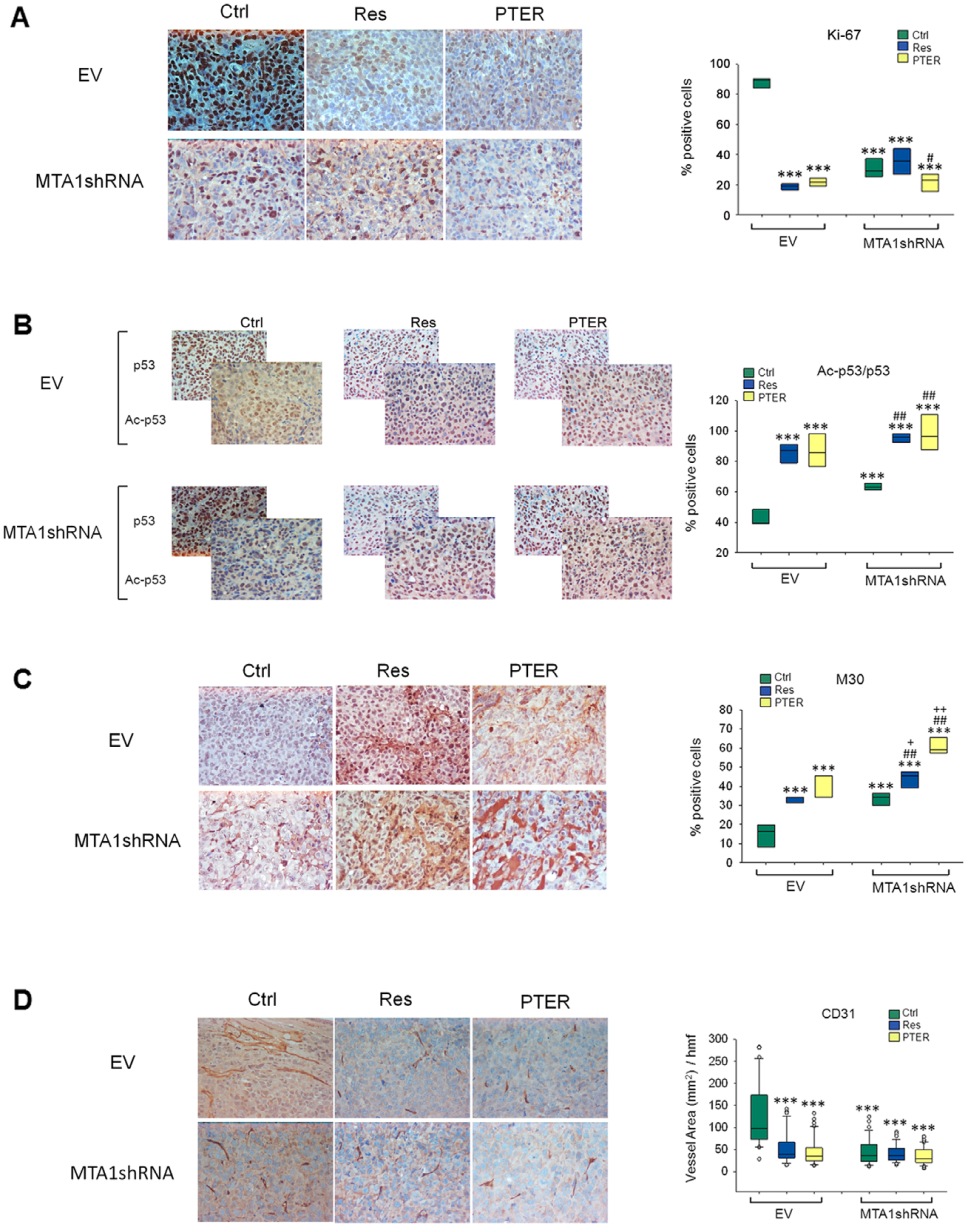
Increased p53 acetylation and apoptosis and decreased angiogenesis by resveratrol and pterostilbene are linked to MTA1 inhibition. *Left,* representative IHC images of A. Ki-67 (proliferation); B. Ac-p53/p53 (MTA1 signaling); C. M30 (apoptosis); and D. CD31 (angiogenesis) in EV- and MTA1shRNA-tumors are shown. *Right,* quantification of positively stained cells as percentage of total cells. Data are box plots of randomly chosen five fields analyzed independently by two investigators. Pairwise comparisons, *p<0.05; **p<0.01; ***p<0.001 vs. EV-Ctrl; ^#^p<0.05 and ^##^p<0.001 vs. MTA1shRNA-Ctrl; +p<0.05 and ++p<0.001, of compounds between EV and MTA1shRNA groups.

To examine the MTA1-dependent effects, we utilized our previously described and characterized Du145 cells transduced with lentivirus carrying EV and MTA1shRNA [13], [19]. We labeled these cells with luciferase and used them for orthotopic inoculation (Fig. 4). Prior to transplantation, validation of luciferase expression and MTA1 knockdown in Du145-Luc cells was performed using bioluminescent assay *in vitro* and Western blot, respectively (Fig. S1). At surgery, mice were injected into the prostate gland with EV-Luc and MTA1shRNA-Luc cells. To assure specific response to MTA1-knockdown and treatment with compounds, we allowed tumors to colonize and develop for 14 days prior to randomization. Tumor development was monitored by BL imaging weekly. Mice that developed tumors (12/16 EV group and 15/16 MTA1shRNA group) were randomized by the size of images in three subgroups and treated with vehicle control (10% DMSO), resveratrol or PTER, i.p., at the same 50 mg/kg/day dose. Pterostilbene dose was kept identical to resveratrol’s to determine potency differences. While vehicle-treated mice showed rapid tumor progression in EV xenografts, resveratrol and PTER caused noticeable delay in tumor growth (Figs. 4A and B). Resveratrol, but more so PTER, showed tumor inhibitory effects, although statistical significance was not reached due to small number of mice. Xenografts expressing MTA1shRNA exhibited markedly reduced tumor growth at week 5 post-transplantation with marginal significance versus EV-Ctrl (p = 0.05). Moreover, MTA1-knockdown tumors treated with compounds had further response, and the differences versus EV-Ctrl became highly significant (p = 0.001 for resveratrol, p = 0.0004 for PTER). Therefore, MTA1-knockdown sensitized cells to resveratrol and particularly to PTER. Consistent with the potent antitumor effects, resveratrol- and PTER-treated tumors showed reduced mitotic activity compared to vehicle-treated EV-tumors as shown by Ki-67 IHC (Fig. 5A). Importantly, consistent with reduced MTA1 activity in tumors, downstream acetyl-target of MTA1, Ac-p53, had significantly increased levels upon treatments, best seen with PTER (Fig. 5B). Accordingly, M30 staining revealed a large increase in apoptosis in tumors from EV compound-treated mice and MTA1-knockdown group (Fig. 5C). Additionally, microvessel area, as assessed by CD31 immunostaining, decreased by ∼59% in EV-compound treated tumors and by ∼67% in MTA1-knockdown group compared to EV-Ctrl (Fig. 5D).

**Figure 6:**
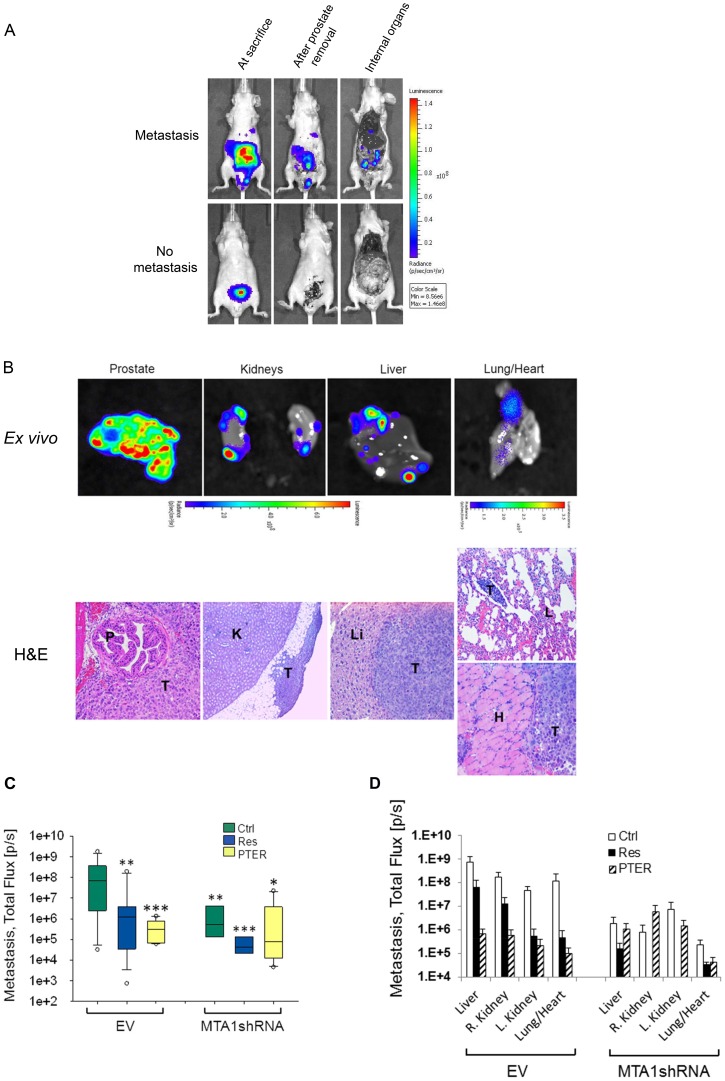
Effects of resveratrol and pterostilbene on spontaneous metastasis: involvement of MTA1. A. BL images of metastasis are shown. Signals detected after prostate removal consisted of metastatic and non-specific signals. Removal of skin and muscles eliminated non-specificity. B. *Top, ex vivo* images of metastatic organs. *Bottom,* Validation of metastatic lesions (T) in kidneys (K), liver (Li) and lung/heart (L/H) by H&E staining. C, quantitative analysis of total metastatic Luc signals in Total Flux (photons/sec/cm2/sr). Open circles represent outliers. *p<0.05; **p<0.01; ***p<0.001 are pairwise comparisons vs. EV-Ctrl. D, quantitative analysis of organ-specific metastasis calculated by luciferase signals as Total Flux (photons/sec/cm^2^/sr). Color-coded histograms of signals for each group are shown. PTER was more effective in inhibiting metastasis in all organs compared to Res in EV-group. In MTA1-knockdown group, Res exhibited more effects by eliminating kidney metastasis.

Few preclinical PCa xenograft models progress to metastasis, making it difficult to study the functional significance of MTA1 in metastasis. Our previous study with LNCaP-Luc cells showed no metastasis when orthotopically injected into mice (unpublished data). Because of the more aggressive phenotype of Du145 cells, we considered the possibility of spontaneous metastasis in orthotopic Du145 xenografts. At the beginning of week 7, we detected BL signals distinct from the primary source (prostate) in sham EV-Ctrl group (Fig. 4A and 6A). *Ex vivo* BL images of metastasis were detected at necropsy in the liver, kidneys and lung/heart, and histology was confirmed by a certified pathologist (JRL) (Fig. 6B). The highest incidence of metastasis was observed in the EV group (100%), whereas the MTA1shRNA group showed only 50–75% and with high degree of heterogeneity (Fig. 6C). Extensive macrometastases in all organs observed in EV-Ctrl were inhibited by resveratrol and PTER. MTA1-knockdown tumors developed smaller metastases, and in fewer organs. Notably, MTA1shRNA tumors treated with compounds either did not develop any kidney metastasis or had small lesions in one kidney (Fig. 6D). The inhibition of metastasis in response to MTA1-targeting agents and MTA1 silencing indicates MTA1 contribution to local invasion, dissemination and metastasis. Together these data suggest that the MTA1 signaling plays an essential role in the development and progression of metastatic prostate cancer and that targeting MTA1 pathway by dietary polyphenols may be effective in slowing down tumor progression and preventing metastasis.

We next measured steady-state serum levels of resveratrol and PTER following 5 weeks of continuous treatment and found that serum accumulation of PTER was always higher than resveratrol (EV: 50 vs. 23.5 ng/ml and MTA1shRNA: 89.1 vs. 52 ng/ml). Interestingly, it appeared that the level of both compounds were higher in MTA1-knockdown group indicating faster rate of elimination in EV group.

## Discussion

Metastasis-associated protein 1 is a critical oncogenic protein and its overexpression correlates with parameters of aggressive tumors: higher grade, angiogenesis, and poor prognosis [14]–[19]. Data from our group and others have shown that MTA1 can be considered as a potential prognostic biomarker for aggressive forms of PCa [15], [19]. We also characterized the role of MTA1 in angiogenesis *in vitro* and in subcutaneous xenografts [19]. No pharmacological agents are known to regulate MTA1 except our own finding on MTA1 regulation by dietary resveratrol [13]. We have previously reported that resveratrol antagonized the ability of MTA1 to inactivate p53-mediated transactivation. In this study, we asked the question whether resveratrol analogues would also inhibit MTA1 and demonstrate higher anticancer potency in animal models.

Pterostilbene (*trans*-3,5-dimethoxy-4′-hydroxystilbene), a dimethylether analogue of resveratrol, naturally found in blueberries, was the most potent MTA1 inhibitor in all cell lines. Interestingly, this effect of PTER was highly target-specific since in growth inhibitory assays 3M-Res demonstrated the most potent antiproliferative effects in LNCaP and Du145 cells while PTER showed the greatest activity in aggressive PC3M cells (data not shown). It was apparent from our studies that replacement of all three hydroxy substituents with methoxy or acetyl groups resulted in considerable growth inhibition of PCa cells. Given that MTA1 is not directly associated with cell proliferation but rather involved in processes associated with metastasis, such as invasion and angiogenesis, it is intriguing that PTER exhibited both growth- and MTA1-inhibitory effects selectively in metastatic PC3M cells, which express the highest MTA1 levels among tested cells (Fig. 2A). Importantly, PTER was more potent than resveratrol in mediating an increase in p53 acetylation through inhibition of MTA1.

To elucidate the MTA1-mediated mechanisms of resveratrol and PTER *in vivo* we utilized PCa orthotopic xenografts with spontaneous metastasis. We showed that both compounds inhibited tumor growth, progression and metastasis. Moreover, our data showed involvement of MTA1-mediated signaling in cancer cell growth and metastasis. We demonstrated that resveratrol, and especially PTER, not only had effects on proliferative and apoptotic indexes but also modulated a specific MTA1-mediated endpoint marker such as Ac-p53. The Ac-p53 to p53 ratio was significantly higher in the MTA1-knockdown group and tumors treated with compounds compared to EV-Ctrl untreated xenografts. MTA1-knockdown sensitized cells to resveratrol and PTER. Although the differences between acetylated p53 in EV and MTA1-knockdown compound-treated tumors were not statistically significant, PTER in combination with MTA1-knockdown demonstrated the most potent apoptotic effect *in vivo* (Fig. 5C). This suggests that near-complete inhibition of MTA1 activity would have remarkable anticancer effects, and that additional compounds, which inhibit MTA1 with even higher potency should be sought, especially for therapeutic uses. Partial inhibition of MTA1 with dietary compounds may be sufficient to slow tumor progression and prevent clinical manifestations of PCa in some patients. Our study is the first *in vivo* validation of the novel MTA1-mediated epigenetic mechanism of PTER that showed the highest apoptotic response in MTA1-knockdown tumors.

We detected higher serum levels of PTER compared to resveratrol in both EV and MTA1-knockdown groups. Previous studies have also shown higher bioavailability for PTER [40]–[43] which was attributed to the replacement of two hydroxy by methoxy groups making PTER more lipophilic and more stable [44]. In addition, PTER’s half-life is several times longer than resveratrol’s [45]. Better bioavailability of PTER could explain its greater *in vivo* activity compared to resveratrol observed by us and others [46]. In the latter study, authors compared the anticancer effects of PTER and resveratrol in colon cancer *in vivo* and found PTER to be a more potent anticarcinogen and anti-inflammatory agent [46]. Pterostilbene has been shown to inhibit micrometastasis in colon, breast, melanoma, pancreatic and hepatocellular carcinoma [43]. In PCa, the anticancer activity of PTER has been shown *in vitro* using LNCaP and PC3 cells [33], [47]. Our current study is the first to demonstrate *in vivo* anticancer and antimetastatic effects of PTER in PCa.

In conclusion, we demonstrated that dietary stilbenes are effective regulators of MTA1/NuRD mediated p53 acetylation, apoptosis and angiogenesis in PCa xenografts. This novel epigenetic signaling provides the first mechanistic evidence of MTA1 as a potential therapeutic target in PCa. MTA1 has the additional advantage of being sensitive to pharmacologically safe dietary compounds. We propose, for the first time, on the basis of strong pre-clinical evidence, that PTER is a lead compound for potent target-specific treatment of MTA1-overexpressing advanced PCa. Our findings substantiate new approaches in PCa management with the inclusion of natural product drugs not only for primary chemoprevention [48] but for anticancer and antimetastatic therapy.

## Supporting Information

Figure S1 **Validation of Luc expression and MTA1 knockdown in Du145-Luc cells.** *Top*. *In vitro* demonstration of differences in Luc-activity of EV and MTA1-knockdown cells further used *in vivo*. Luc-positive cells were tested to confirm specificity and sensitivity. MTA1-knockdown-Luc cells showed greater Luc activity when normalized with EV-Luc cells. Negative controls: media only and untransformed cells. *Bottom,* stable MTA1 knockdown in Du-145-Luc cells was confirmed by Western blot three days before transplantation into mice.
(TIF)
