# Supplementary figures and images for: Pterostilbene Acts through Metastasis-Associated Protein 1 to Inhibit Tumor Growth, Progression and Metastasis in Prostate Cancer
Source: PLoS One. 2013 Mar 1;8(3):e57542. doi: 10.1371/journal.pone.0057542 (PMC3586048; doi:10.1371/journal.pone.0057542)

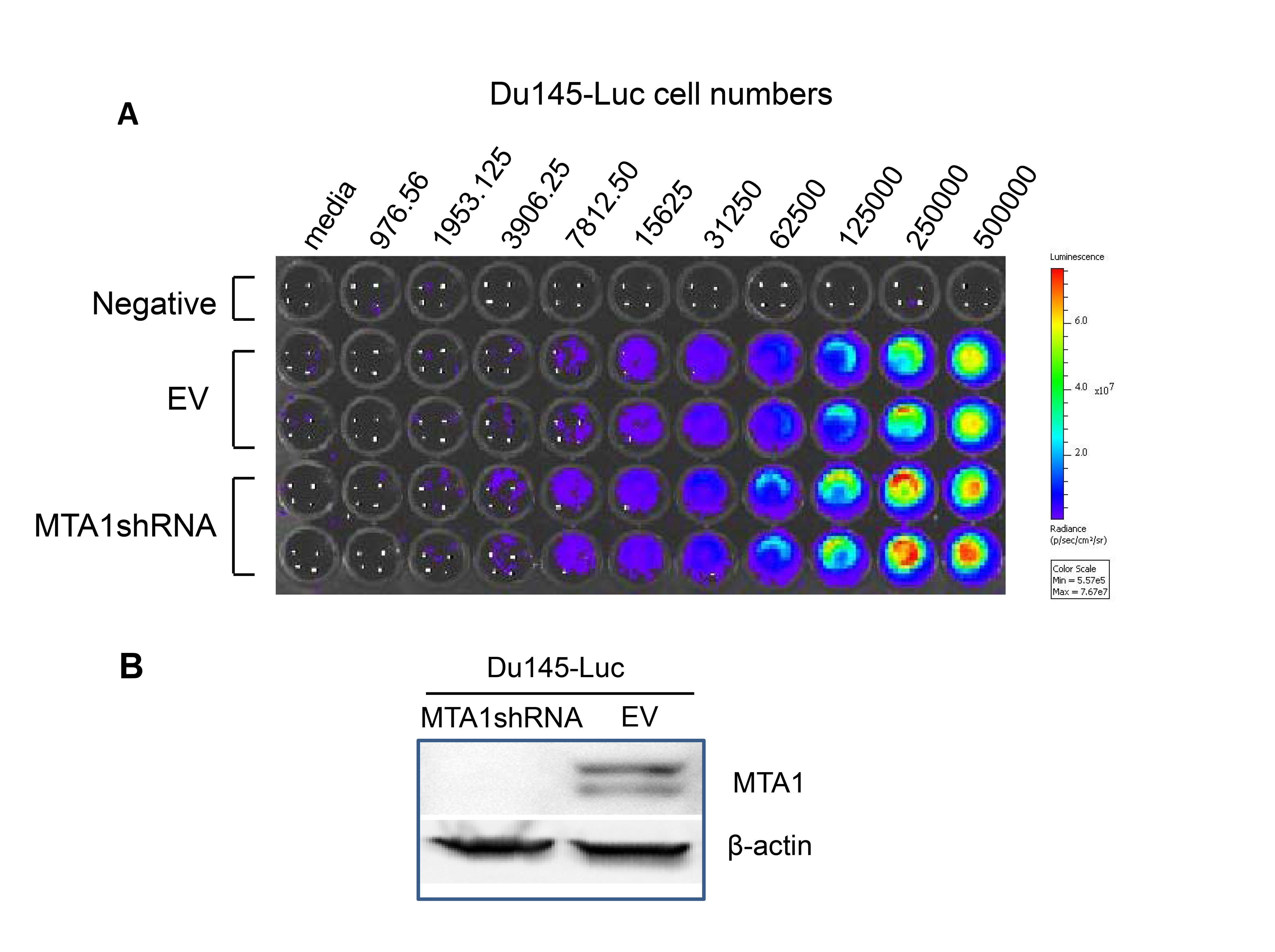

Supplement: Figure S1 — Validation of Luc expression and MTA1 knockdown in Du145-Luc cells. Top. In vitro demonstration of differences in Luc-activity of EV and MTA1-knockdown cells further used in vivo. Luc-positive cells were tested to confirm specificity and sensitivity. MTA1-knockdown-Luc cells showed greater Luc activity when normalized with EV-Luc cells. Negative controls: media only and untransformed cells. Bottom, stable MTA1 knockdown in Du-145-Luc cells was confirmed by Western blot three days before transplantation into mice. (TIF) [file pone.0057542.s001.tif]
